# Quartzite Mining Waste: Diagnosis of ASR Alkali-Silica Reaction in Mortars and Portland Cement Concrete

**DOI:** 10.3390/ma14247642

**Published:** 2021-12-11

**Authors:** Ivan Francklin, Rogério Pinto Ribeiro, Fernando Augusto Corrêa

**Affiliations:** São Carlos School of Engineering, University of São Paulo—USP, São Paulo 13566-590, Brazil; ivan.francklin35@gmail.com (I.F.J.); fernandoaugustocorrea@usp.br (F.A.C.)

**Keywords:** foliated quartzite mining, aggregate, ASR, concrete durability and microstructure, sustainable development

## Abstract

The main objective was to determine the deleterious potential of quartzite mining tailings subjected to different ASR alkali–silica reaction tests. The studies included petrographic analysis, chemical analysis of cements, expansion tests in mortar bars and concrete prisms, and microstructural analysis. Petrographic analysis of quartzites indicated high percentages of deformed quartz (95%), and were classified as potentially reactive. Two types of HES high early strength cement with alkaline equivalents of 0.749% and 0.61%, respectively, were selected. Of the 8 samples analyzed by the accelerated method in mortars, only 2 quartzite samples and 1 diabasium sample indicated potentially reactive behavior. The accelerated and long-term methods in concrete prisms proved to be effective and were consistent with the deleterious potential of the samples. All analyzed samples were diagnosed with the ASR gel. In the microstructural analysis, in addition to the ASR products, other expansive products of late ettringite were detected. Reaction mitigation methods are proposed so that quartzite waste can be used as an alternative aggregate in concrete, and thus contribute to the reduction of mine tailings and, consequently, reduce the negative environmental impact from mining.

## 1. Introduction

Brazil is one of the world’s largest producers and exporters of dimension stones. The state of Minas Gerais ranks second in the production of these materials, according to recent data [1], representing about 20% national market with 1,800,000 tons produced in 2020, referring to a wide variety of rocks (granite, pegmatite, slate, foliated and massive quartzite, soapstone, among others). As far as foliated quartzites are concerned, Minas Gerais accounts for most of the Brazilian production of stone cladding, which is mainly found in four regions of the state: São Thomé das Letras, Alpinópolis, Ouro Preto, and Diamantina [2], with the first two being the most representative. These quartzites, also called flagstones (“pedra mineira”), are used as decorative elements in civil works, and are basically produced as slabs, fillets and small prismatic fragments used in the execution of mosaics.

**Figure 1:**
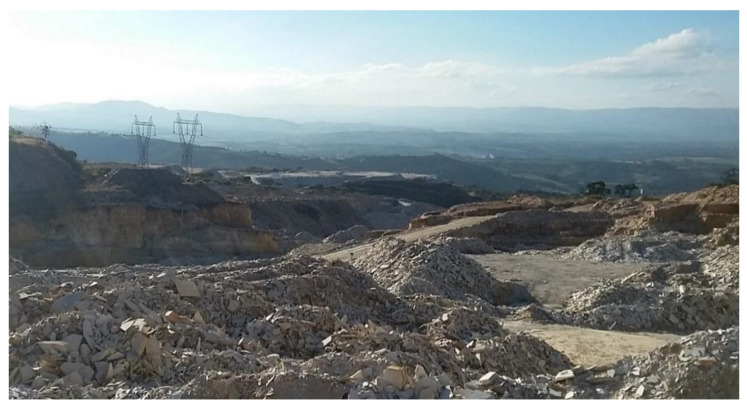
Example of large volumes of quartzite mining tailings.

Waste generation is inherent to any process of production or transformation of materials and, in the context of sustainable development, more in-depth studies on waste have been intensified [3,4,5,6]. In the stone industry, for example, different types of waste are produced from extraction from the rock mass to the final operations for the production of slabs with commercial dimensions [7,8,9]. In the case of quartzite mining, large volumes of waste are generated, which may exceed 90% of the exploited material (Figure 1). This is because foliated quartzite slabs are obtained directly from the rock mass, following standards of thickness and length, and the waste generated in mining and processing of these materials becomes a problem for entrepreneurs, with negative environmental impacts, such as: landscape disfigurement; changes in the natural shape of the relief; siltation of water bodies; supply for the native vegetation and instability on slopes of mine waste deposits [10], in addition to causing public health problems, since silica particles are released into the atmosphere that, when inhaled, can cause respiratory damage [11]. These problems (or some of them) should be similar in quartzite and slate (foliated rocks) quarry activities in other regions around the world, notably in the main countries producing dimension stones.

In this context, several geotechnical studies have been conducted with the aim of making quartzite mining tailings an alternative source of raw material for different purposes in engineering works or civil construction, for example, aiming to use this waste as aggregate in concrete [12,13,14]. Most studies involved the production of structural concrete, with the need to carry out durability studies on these products, given the possible expansive reactions that may occur between reactive minerals and chemical components of Portland cement. Rangaraju et al. [15] highlight the importance of these studies, especially when minerals are not added to concrete composition. One of the pathological phenomena occurring in silica-rich rocks is the AAR alkali–aggregate reaction and is addressed in this article given the strong evidence of the damaging capacity of quartzites in contact with alkaline cements and in aggressive environments. Alkali–silica potential reactivity is one of the reactions from AAR, and occurs when combining water, cement alkalis (Na and K) and aggregate silicas, and can produce an expansive gel inside the concrete structure [16]. This type of pathology has been extensively studied for a better understanding and interpretation of the forms of pathological manifestations through the development of different diagnostic methods of ASR alkali–silica reaction as presented by Strack et al. [17], Sachlová et al. [18] and Tiecher et al. [19], among other examples.

Studies carried out by Pinheiro [20] and Collares et al. [12], using quartzites from the southeast region of the state of Minas Gerais, demonstrated a potentially deleterious behavior in the samples, however these studies were limited to using only the accelerated test following the procedures established by ASTM C1260 [21]. Another investigation was also performed by Hasparyk et al. [22] on concrete structures of a hydropower plant built in the 50′s using these quartzites and cements with high alkali (Na and K) content. According to the aforementioned authors, the formation of the expanding gel (pathology) only occurs when the concrete structure was in direct contact with water, with the formation of the exudate gel, detected in some points of the dam. These authors also proposed preventive and inhibitory methods of the reaction for the non-occurrence of structural damage.

Based on these premises, the general objective of this study was to identify the degree of deleterious potential of quartzite tailings from Minas Gerais in mortars and concrete attacked by alkaline solutions. The specific objectives are: to identify and classify the reactive minerals in different types of quartzite tailings through petrographic analysis; to determine the chemical composition and calculate the equivalent alkali in samples of Portland cement; to classify the deleterious potential of quartzites by means of laboratory expandability tests; and to investigate the presence of pathological products in the microstructure of mortars and concretes using SEM scanning electron microscopy.

## 2. Description of the Study Area and Material

Samples of quartzite mining wastes used here were selected and collected from mining waste dumps located in the municipalities of STL São Thomé das Letras and ALP Alpinópolis, including previously crushed quartzites, and classified by a sector company. In the present study, a diabase exploited in a quarry located in the region of SSP São Sebastião do Paraíso was the reference rock for comparative purposes, as it is a material widely marketed as aggregate for concrete in the southwest region of the state of Minas Gerais, Brazil.

Field surveys and preliminary recognition of the geological-geotechnical framework of the main exposures of quartzites mined in the STL and ALP regions, combined with the empirical denomination of miners to classify the waste, detected differences in tonality and texture of the materials according to the extraction site, and three geotechnical types of quartzites were categorized, commercially called soft, hard and glazed. The characteristics of these different types of quartzite tailings and the conventional diabase aggregate are listed in Table 1.

## 3. Experimental Program

Table 2 presents a summary of the test procedures and the respective technical standards used.

Importantly, each of the established test methods sets procedures with different temperatures, as the higher the temperatures, the more intense the chemical reactions between the alkaline components of the cement and the silica in the aggregates. The accelerated mortar bar method [25] uses a temperature of 80 °C [11,12,17,18,28], allowing to obtain results in only 30 days of tests. The long-term concrete prims method [26] uses a temperature of 38 °C [6,29], but with a duration of 1 year. In turn, the accelerated concrete prism method [27] uses a temperature of 60 °C [27,30,31,32] and allows obtaining results with testing for a minimum of 20 weeks.

### 3.1. Chemical Analysis of Portland Cement

The two types of HES Portland cement were chosen for technological studies as they do not contain pozzolanic additions and steel slag in their compositions, which can contribute to ASR mitigation. In this way, there is a natural tendency for greater alkalinity in composition. Chemical analyses were performed to obtain the alkali content (Na and K) in the cement (Table 3). Studies carried out by Tiecher et al. [33] with aggregates from southern Brazil combined with different types of cements, proved the most unfavorable situation of rapid hardening cement in promoting the formation of the expanding gel in mortar bars subjected to expansion tests using the accelerated method. On the other hand, there are several products that mitigate this reaction, such as the use of silica fume and other pozzolanic products (blast furnace slag, fly ash, among others) that are proven to be effective in reducing the equivalent alkali in mortars and concretes [28,34].

Both technical standard recommendations and recommendations from several researchers [17,18,22] highlight the importance of analyzing the ASR reactivity in silica-rich aggregates (as is the case of quartzites) combined with alkaline cements free of mineral additions, such as the CP V-ARI. This cement was adopted in the present study because it is vulnerable to attack by the ASR and also because it is widely consumed in civil construction.

### 3.2. Mortar Bar Expansion by the Accelerated Method

**Figure 2:**
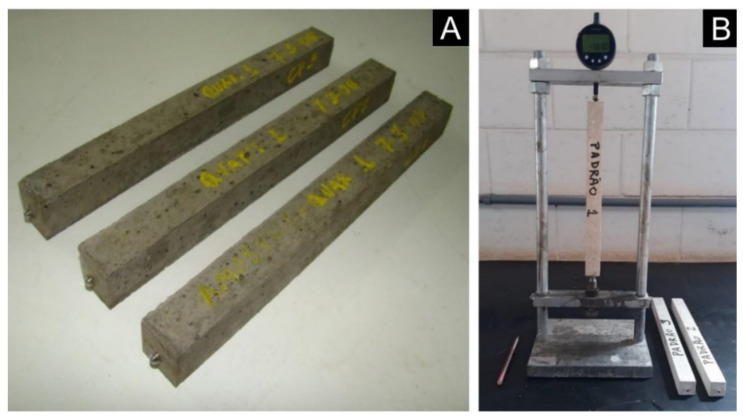
(**A**) Mortar bars; (**B**) Expansion measurement device.

The accelerated test method, recommended by NBR 15577-4 [25] and ASTM C1260 [21], determines through variation in length (expansion) of mortar bars (Figure 2) the susceptibility of an aggregate to participate in the alkali–silica expansive reaction in the presence of hydroxyl ions combined with cement alkalis. Three mortar bars were produced in the dimensions of 25 × 25 × 285 mm^3^ with a fixed mix ratio at 1:2.25:0.47 for each of the eight samples of aggregates and subjected to alkali attack in a NaOH solution at a temperature of 80 °C.

After casting, bars remained at rest in a humid chamber. After the curing period, bars were removed from the molds, identified and the initial reading was made. Afterwards, bars were submerged in water and kept in an oven at 80 °C for 24 h. Then a new reading was taken and the bars were transferred and submerged in the standard solution with 1 N NaOH, and kept in an oven at 80 °C, accelerating the reactions and allowing the identification of results in a short period of time. Expansion readings were taken periodically, at 8, 16, 22 and 30 days, counted from the casting, and the results expressed in percentage of expansion, corresponding to the means of the three bars. For expansions of up to 0.19%, at 30 days, the aggregate was classified as potentially innocuous, and for expansions greater than 0.19%, as potentially reactive.

During the production of some mortars, friability was observed in quartzite samples STL1, ALP1 and ALP3, which promoted the self-fragmentation of the materials during mixing in the mortar mixer. In the specific case of the ALP1 sample, in addition to altering the particle size of the aggregate, self-fragmentation resulted in an increase in the specific surface area, requiring a superplasticizer additive to promote the fluidity necessary for casting the bars.

### 3.3. Concrete Prism Expansion by Long-Term and Accelerated Methods

**Figure 3:**
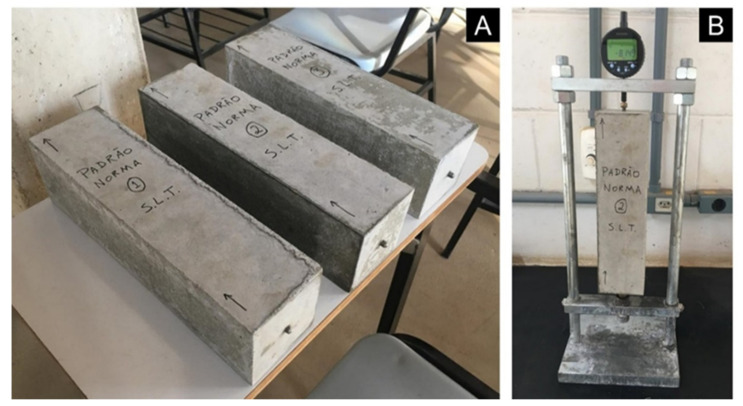
(**A**) Concrete prisms; (**B**) Expansion measurement device.

For the alkali–aggregate potential reactivity test by the long-term and accelerated methods in concrete prisms, the procedures established by NBR 15577-6 [26] and by NBR 15577-7 [27] were adopted, respectively. Concrete dosages followed: cement consumption in 420 kg/m^3^ concrete; the water/cement ratio at 0.45; the dry coarse aggregate compact volume by 70%; the dry mortar content by 50%; and fineness modulus of fine aggregate at 2.7. Table 4 presents the relationship of samples and mixes produced. Concretes were produced with innocuous fine aggregates and with quartzite coarse aggregates, according to the normative standard NBR 15577-1 [35]—STL0 St standard, and concretes with fine and coarse quartzite aggregates—STL0 100%. Concrete consolidation in prismatic molds was carried out in three layers with the aid of a flow table. After casting, prisms were placed in a humid chamber for 24 h, and initial reading was taken (Figure 3). Subsequently, prisms were removed from the oven and placed in a humid chamber at 23 ± 2 °C, before each reading. This was necessary because significant divergences in expansion may occur if these time and temperature intervals are not respected.

To determine expansion by the long-term method, concrete prisms were kept in a temperature-controlled environment (38 ± 2 °C). The expansion readings were taken at 7, 28, and 56 days, followed by monthly readings from 3 to 12 months. Equivalent alkali (Na_2_Oeq) of the cements used to calculate and adjust the contents to 1.25% were made by adding NaOH to the concrete mixing water, using a conversion factor. STL3, ALP1 and ALP3 samples proved to be quite friable during concrete mixing and consolidation. The particles fragmented up, thus changing the particle size of the materials, and consequently the concrete consistency due to the increased specific surface of the aggregates. As an alternative to facilitate prism consolidation, without changing the w/c ratio, a superfluidizing additive was added to the mixtures. The ALP1 sample, even with the addition of the additive, was discarded for presenting a high porosity index and not meeting the consolidation conditions.

The accelerated prism method was standardized by ABNT in 2018, and several studies contributed to the establishment of this procedure, such as Wallau et al. [30] and Sanches et al. [31]. The study carried out by Shi et al. [32] points out good contributions to the accelerated method in prisms when using temperatures of 80 °C. In the range between 60 °C and 80 °C, the crystalline ASR products formed in concrete when exposed to KOH, NaOH solutions and the KOH/NaOH mixture that contribute to the interpretation of microstructural features of mortars and concretes were investigated. To determine the expansion by the accelerated method, in addition to the expansion reading with the aid of a comparative gauge, concrete prisms were measured in length with the aid of a caliper. After determining the initial 24 h expansion measures, specimens were placed upright in an airtight container and subsequently taken to an oven (60 ± 2 °C). Length variation readings were taken at ages 5, 10, 15, 20, 25 and 30 weeks.

### 3.4. Microstructure Analysis by SEM Scanning Electron Microscopy

According to Mohammadi et al. [29], in some rock types, such as quartzite, for example, gel formed by ASR generally occurs on the aggregate surface, forming products rich in silica and calcium. Alkalis usually migrate through surface layers faster than calcium, facilitating aggregate decomposition and ASR gel formation. This cycle is repeated and leads the concrete to expansion through internal tensile forces in the ITZ interfacial transition zone, between the aggregate and the cement matrix. Strack et al. [17] identified expansive products from the ASR using SEM in mortars made with quartzite aggregates (Las Placitas), from the USA, previously attacked with NaOH and KOH solutions.

In this study, SEM photomicrographs of mortar bars and concrete prisms were obtained using the ZEISS LEO 440 equipment. Fragments selected for microstructure analysis were taken from strategic points in the samples, which presented significant expansions during expansion tests and that showed cracks on their surfaces. Analyses were performed using the SE secondary electron detector to obtain the images and EDS energy-dispersive spectroscopy for microanalysis of the products selected for investigation.

## 4. Results and Discussion

### 4.1. Petrographic Analysis of Quartzites

**Figure 4:**
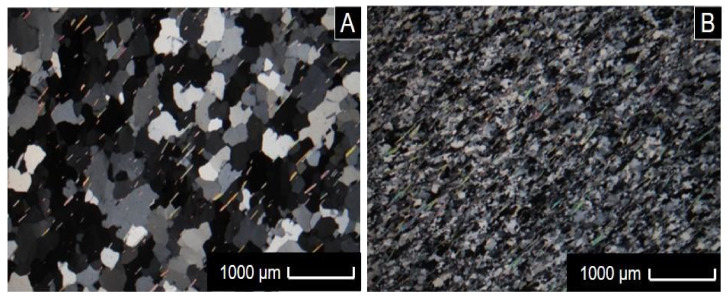
Photomicrographs (crossed nicols). (**A**) STL quartzite showing the granoblastic arrangement of quartz crystals and foliation marked by the alignment of muscovites; (**B**) ALP quartzite with the same textural arrangement but with considerably smaller grain size.

Quartzite samples were subjected to petrographic analysis using a transmitted light petrographic microscope. Results in Table 5 indicated small mineralogical variations of muscovite between samples of STL quartzite tailings. Microscopically, they were classified as foliated quartzites with fine muscovite, with a strongly anisotropic structure with well-oriented muscovite and grano-lepidoblastic texture, consisting of approximately 94.5% quartz and the remainder (5.5%), of muscovite, opaque minerals and tourmaline (Figure 4). Comparatively, APL samples were considerably thinner than the STL. The average particle size of STL was predominantly between 0.1 and 0.4 mm in width and about 0.2 and 0.6 mm in mineral stretching, while the average particle size of ALP was predominantly between 0.01 and 0. 04 mm in width and about 0.06 and 0.1 mm in mineral stretching. Regarding the cohesion of the rocks, in general the STL muscovite quartzites were considerably more cohesive (less friable) than the ALP. This is due to the better recrystallization of the STL muscovite quartzites, considering that they are rocks with a metamorphic degree considerably higher than ALP rocks. Another factor to take into account regarding cohesion is the weathering intensity of each set of samples. In this case, the STL1 sample demonstrated the greatest weathering effects in its set, which would make it more friable than the others.

Chiodi Filho et al. [36] carried out a petrographic and mineralogical characterization of different types of quartzites in sixteen samples from five quarries in the STL municipality, with quartz contents ranging from 95% to 98% and muscovite contents ranging between 3% and 5%. These are percentages of the predominant mineralogical constituents (quartz and muscovite) similar to the quartzites analyzed herein, stating that the STL and APL quartzites were practically identical in terms of mineralogical aspects.

### 4.2. Expansion in Mortar Bars by the Accelerated Method and SEM Analysis

**Figure 5:**
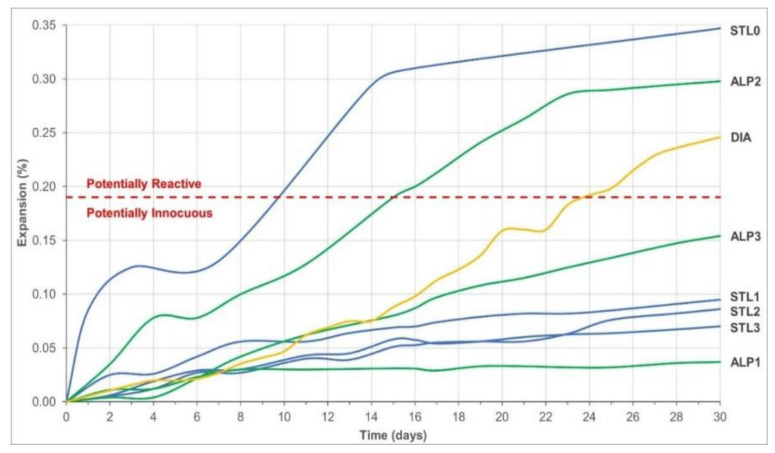
Summary of expansion test results by the accelerated mortar bar method.

Figure 5 illustrates a comparative overview of the average expansion of mortar bars up to the age of 30 days, tested by the accelerated method, and only the STL0 and ALP2 quartzite samples and DIA diabase showed a potentially reactive behavior, with expansions above the limit of 0.19% established by NBR 15577-1 [35], highlighting the quartzite samples that presented significant expansions in the first days of testing. Thus, to verify the occurrence of the ASR expansive gel, samples showing potentially reactive behavior were selected for microstructural analysis using SEM. Reis et al. [10] also used the accelerated method to analyze the expansion of mortars produced with ALP quartzites. The average expansion obtained was approximately 0.25% at 28 days, therefore close to the result of the ALP2 sample obtained in the present study. Barros et al. [11] studied the potential alkali–aggregate reactivity of quartzite tailings from northeastern Brazil, and reported minor expansions; however, performed only the test in mortar bars and did not investigate the microstructure of the composite. Sachlová et al. [18] analyzed quartzites from Prague-Czech, and verified expansive behavior when performed accelerated tests in mortar bars and proved ASR using SEM.

**Figure 6:**
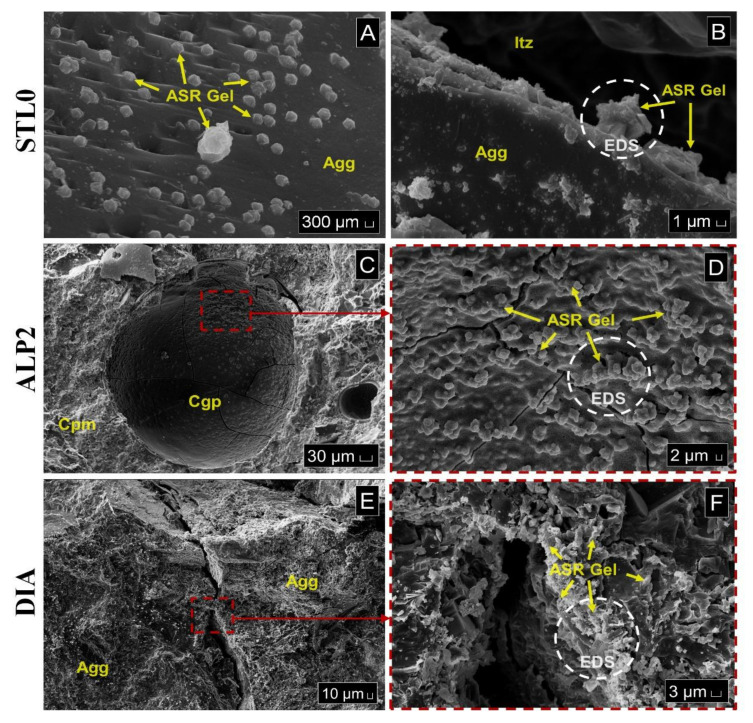
Mortar bar microstructure by SEM analysis. STL0: (**A**) Gels developed and adhered to the edge of the Agg aggregate. (**B**) Gels being formed on the aggregate surface close to the Itz interface transition zone; ALP2: (**C**) Cgp Cracked solid gel products adhered to the pore surface around Cpm cement paste matrix. (**D**) Detail showing the appearance of the cracked and botryoidal gel; DIA: (**E**) Fracture interface where the investigations were carried out. (**F**) Gel deposits on the aggregate surface caused by ASR.

**Figure 7:**
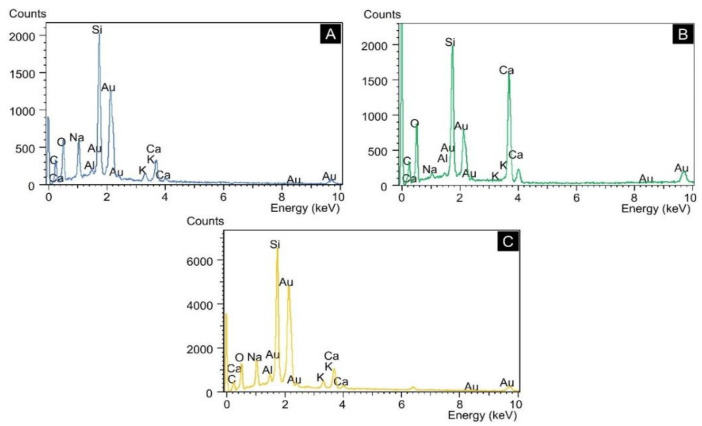
EDS analysis on ASR products. (**A**) STL0; (**B**) ALP2; (**C**) DIA.

During the microscopic investigations and the EDS analysis performed on mortar bars, it was possible to observe in the STL0 sample, characteristic products of crystallized gels adhered to the edge of the aggregate and the typical generalized formation of ASR silica gels on the aggregate surface (Figure 6A,B). In the ALP2 sample, a pore with the appearance of a solid cracked gel was identified (Figure 6C) and, after image magnification, the cracked fissures and the formation of botryoidal gel, typical of ASR, were verified (Figure 6D). The EDS spectrum analysis showed that the crystalline products generated on the aggregate surface are ASR silica gels (Figure 7A,B). The DIA diabase microstructure images were collected inside the fissure region in the sample, where the widespread presence of crystallized products was found (Figure 6E,F). These crystals were subjected to EDS analysis and the hypothesis of the formation of the ASR silico–calco–alkaline gels was confirmed (Figure 7C).

### 4.3. Expansion in Concrete Prisms by the Long-Term Method

**Figure 8:**
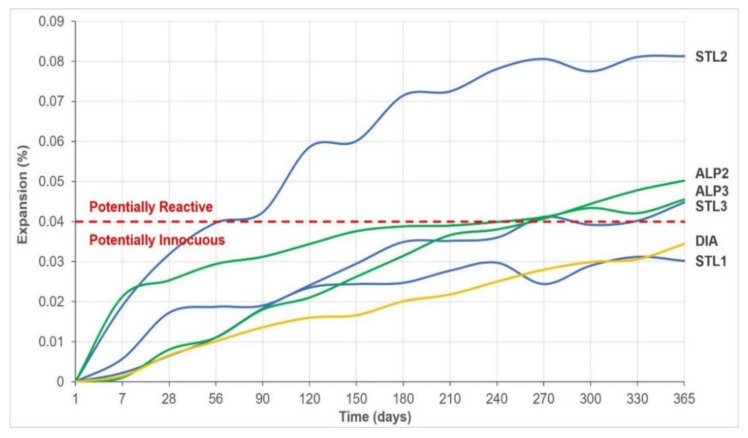
Synthesis of the results of the long-term expansion test on concrete prisms.

Figure 8 presents the summary of the average expansion results by the long-term method in concrete prisms, up to the age of 365 days. The STL2 sample showed a potentially reactive behavior, with significant expansion at the very beginning of the test, above the limit of 0.04% [35]. The sample did not present spans, and no signs of fissures were observed on the prism surface, for this reason it was not selected for analysis by SEM. On the other hand, this same sample tested by the accelerated method in mortar bars showed little significant expansion, and was classified as potentially innocuous. It is important to highlight that, according to NBR 15577-1 [35], the results obtained by the long-term method prevail over those obtained by the accelerated method, as it is a more reliable procedure. The results of samples STL3, ALP2 and ALP3 up to the age of 365 days slightly exceeded the limit established by the standard, and were classified as potentially reactive. Sanches et al. [31] carried out long-term studies in concrete prisms using quartzites from the Brazilian Midwest region and reported expansions close to 0.04% at 365 days, therefore similar to the results obtained in the present study.

The average expansion of concrete prisms produced with diabase aggregate showed a potentially innocuous behavior, contrary to the test carried out by the accelerated method on mortar bars, which showed a potentially reactive behavior.

It is important to inform that, in other studies, some methods were tested increasing the analysis time in order to check the expansive behavior in more advanced ages. As an example, Yuksel et al. [37] evaluated concrete prisms with siliceous aggregates (glass residues) and verified the low expansion behavior at 365 days, but with the advancement of analyses up to the age of 1095 days (3 years), these authors found that some samples fell into the class of reactive potential with values above 0.04% expansion.

### 4.4. Expansion in Concrete Prisms by the Accelerated Method and SEM Analysis

**Figure 9:**
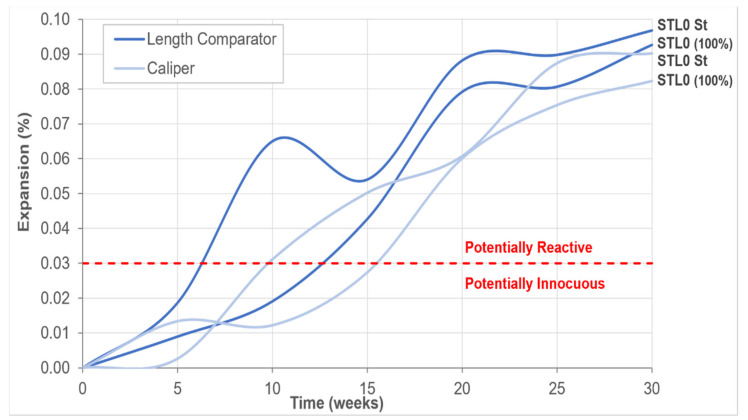
Summary of average expansion results by the accelerated method in concrete prisms.

**Figure 10:**
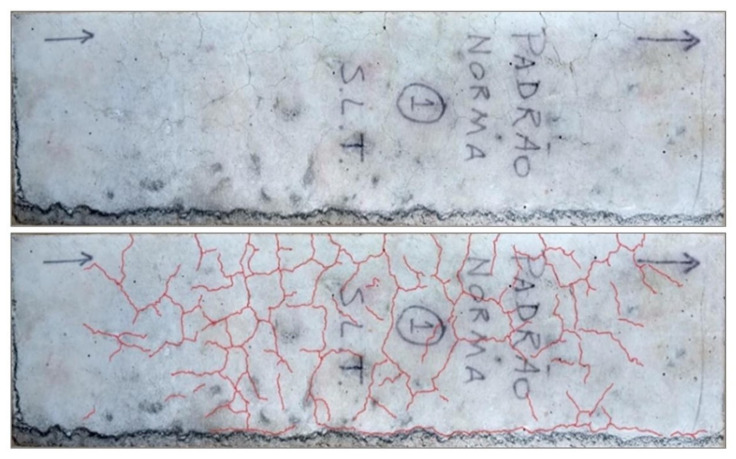
Typical mapped fissures (TOP—real images; BOTTOM—highlighted in red) on a face of the STL0 St prism.

Figure 9 shows the average expansion of two samples, six concrete prisms produced with 100% fine and coarse quartzite aggregate (STL0 100%) and three concrete prisms produced according to the normative standard (STL0 St). Expansion measures of concrete prisms were taken with a length comparator coupled to the measuring apparatus and comparative readings were also taken with the aid of a digital caliper. Although NBR 15577-7 [27] recommends taking the readings until the age of 20 weeks, it was decided to extend the readings until the age of 30 weeks. All analyzed concrete prisms showed a potentially reactive behavior, with expansions greater than 0.03%. The STL0 sample (100%) did not show any spans, and few signs of fissures were observed on the prism surface. The sample expansion results showed similarity with the use of the two types of measurements used. In the STL0 St sample, it was possible to observe narrow spans and generalized cracks on the prism surfaces (Figure 10). The expansion results of this sample, using the two types of measurements, showed divergences up to the age of 20 weeks, but at the ages of 25 and 30 weeks, the results started to look similar, with a progressive growth. For these reasons, samples were subjected to SEM analysis. Studies carried out by Fournier et al. [38],Wallau et al. [30] and Sanches et al. [31], in concrete samples subjected to accelerated testing using different lithological types of aggregates (including quartzites), showed that this method can be considered reliable for the interpretation of alkali–aggregate reactivity, as the results obtained were consistent with the degree of deleterious potential of the analyzed samples.

**Figure 11:**
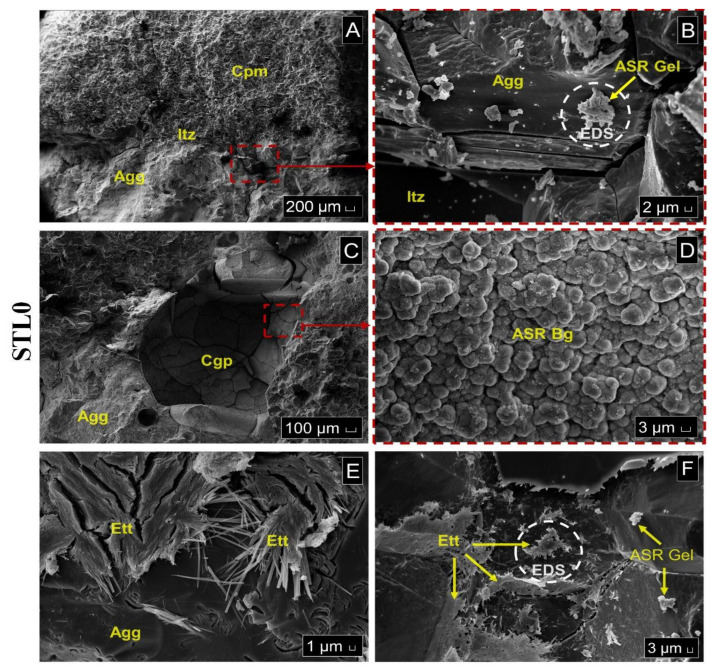
SEM analysis of the microstructure of mortar bars. STL0 St sample: (**A**) Investigated Itz interface transition zone; (**B**) Gel deposits on the Agg aggregate surface caused by ASR; (**C**) Cgp solid cracked gel products adhered to the pore surface; (**D**) Aspect of the Bg botryoidal gel adhered to the pore surface; (**E**) Ett ettringite formation on the aggregate surface. STL0 (100%) sample: (**F**) Typical products of ASR and ettringite formation on the aggregate surface.

**Figure 12:**
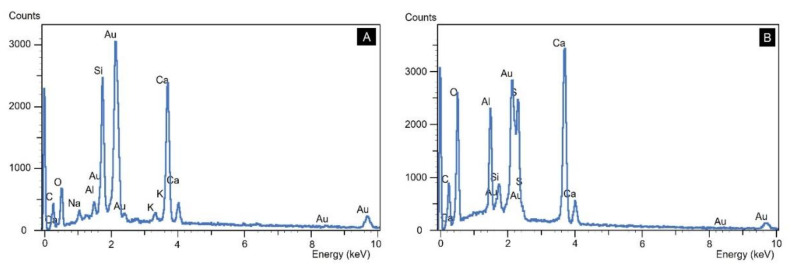
EDX spectroscopy analysis. (**A**) STL0 St; (**B**) STL0 (100%).

Photomicrographs in Figure 11A–E are from the STL0 St sample. During the investigation, the presence of crystallized products of alkaline silico–calcium composition formed on the aggregate were observed (Figure 11A,B). In another analysis, the presence of a solid cracked gel, typical of ASR, was found inside a pore (Figure 11C) and in the same magnified image, crystallized products had a botryoidal appearance (Figure 11D). The EDS spectrum performed inside the pore evidenced that these are crystallized silicon gels from the ASR (Figure 12A). Formations suggestive of DEF delayed ettringite formation adhered to aggregate surfaces were also seen in some points in the ITZ (Figure 11E). In the investigation carried out in the STL0 (100%) sample, it was possible to verify in some points in the ITZ, the presence of crystallized silicon gels and calcium sulfoaluminate products, typical of DEF (Figure 11F and Figure 12B).

Table 6 presents a summary of the results obtained by the expansion methods and the final diagnosis obtained with SEM. All tested samples were diagnosed with the ASR gel. Most samples analyzed by the accelerated method in mortar bars resulted in non-reactive (innocuous) behavior, which proves that this methodology is less effective compared to the long-term method and the accelerated method using concrete prisms, which indicated the reactive potential of the aggregates. Regardless, microstructure analysis using SEM is essential for accurate diagnosis of this pathology.

In view of the above and the evidence of expansions caused by ASR in mortars and concrete produced with quartzites from the southeast region of Brazil, it is recommended mitigating actions for the execution of concrete structures subjected to contact with water. NBR 15577-1 [35] suggests limiting alkalis in concrete and/or the use of suitable inhibitor materials like silica fume, fly ash, metakaolin and cements with addition of slag or pozzolan, for example. Some examples of ASR mitigation studies have shown the efficiency of using fly ash (between 15% and 25%), silica fume (10%) and blast furnace slag (between 40% and 60%) as partial substitutes for cement in concrete produced with aggregates from Australia [34] and Colombia [28]. Alternative mitigation methods have been researched using calcium nitrate [39] and calcium hydroxide [40], showing great effectiveness with the use in glass aggregates. Another proven effective mitigation method is the use of lithium nitrate, which reduces the silica dissolution rate of the aggregates inside the concrete [41,42,43,44].

## 5. Conclusions

This study investigated the reactive potential of quartzite tailings from southeastern Brazil, in mortar bars and concrete prisms subjected to different types of alkali attacks in aggressive environments.

The petrographic analysis on quartzites was important to warn about the risks inherent to ASR in concrete produced with this material, as the percentage of silica obtained in the samples totaled approximately 95%.

The results using the different AAR test methods showed the effectiveness of the accelerated methods in concrete prisms and the long-term tests in concrete prisms, while the accelerated method in mortar bars showed a lower level of reliability.

During the execution of microstructure analysis using SEM equipment, all tested samples were diagnosed with the presence of ASR expansive gels, indicating the need to use this method to perform a precise diagnosis of this pathology. In addition, expansion products typical of DEF developed on aggregate surfaces, which may have been developed with the rise in temperature during the test, added to the concentration of CaSO_4_ (calcium sulphate), were also detected. Specific tests of sulfate attacks are recommended for a better diagnosis of this eventual problem, and we therefore propose mitigation methods for this reaction, such as the use of cement resistant to sulfates, for example.

The diagnosis of AAR in quartzites and diabase corroborated the importance of investigating the chemical reactions resulting from this pathology in concrete produced with these aggregates. In addition, this allows engineers and researchers to direct the correct use of these materials in different concrete structures, and warns against adopting techniques to minimize this reaction in structures that will be in contact with water, such as dams, dikes, bridges and retaining walls.

In this way, quartzite tailings can be used as an alternative aggregate in mortars and concretes, and can contribute to reducing the amount of material destined for mining waste dumps, consequently mitigating the negative environmental impact in the south of the state of Minas Gerais, Brazil.

## Figures and Tables

**Table 1:** Summary of the studied material.

Region (MG)	Samples	Material Description
São Thomé das Letras	STL0	Industrialized Quartzite.
	STL1	Friable, foliated quartzite, with lower apparent mechanical strength.
	STL2	Quartzite with incipient foliation and medium apparent mechanical strength; widely used as a “cladding stone”.
	STL3	Silicified quartzite, solid structure, non-foliated; does not allow exploitation of slabs.
Alpinópolis	ALP1	Quartzite with incipient foliation and medium apparent mechanical strength; widely used as a “cladding stone”.
	ALP2	Silicified quartzite, solid structure, non-foliated; does not allow exploitation of slabs.
	ALP3	Friable, foliated quartzite, with lower apparent mechanical strength.
São Sebastião do Paraíso	DIA	Diabase conventionally used as aggregate for concrete.

**Table 2:** Summary of test procedures and respective standards.

Test	Standard	Basic Description
Petrographic analysis	NBR 15577-3 [23]	Optical microscopy in thin sections obtained from representative fragments.
Cement chemical composition	ASTM C114 [24]	Alkali content (Na and K) and equivalent alkali of HES high early strength Portland cements from the manufacturers Votorantim Cimentos and Lafarge-Holcim, respectively.
Accelerated Mortar Bar Expansion test	NBR 15577-4 [25] C1260 [21]	Production of 3 bars for each of the 8 samples, with dimensions 25 × 25 × 285 mm^3^ and mix ratio 1:2.25:0.47. Expansion readings, periodically, for up to 30 days.
Long-term Concrete Prism Expansion test	NBR 15577-6 [26]	Production of 3 prisms for each of the 8 samples, except for STL0 and ALP1, dimensions 75 × 75 × 285 mm^3^. Cement consumption 420 kg/m^3^; w/c water-cement ratio 0.45; 70% coarse aggregate volume; 50% dry mortar content; FM fineness modulus in 2.7. Expansion readings, periodically, up to 365 days.
Accelerated Concrete Prism Expansion test	NBR 15577-7 [27]	Production of 3 prisms with “standard mix” (STL0 St) and 6 prisms with 100% quartzite (STL0 100%). Dimensions 75 × 75 × 285 mm^3^; Expansion readings using comparative gauge and a caliper, periodically, for up to 20 weeks.
Microstructural analysis	Non- Normative	SEM photomicrographs were obtained in a ZEISS LEO 440 equipment. Samples were coated with 6 nm gold and kept in a desiccator until analysis. Finally, samples were subjected to EDS energy-dispersive spectroscopy analysis.

**Table 3:** Specification of HES Portland cements used in the present study and their respective equivalent alkali.

Type	Manufacturer	Lot/Manufacturing	Equivalent Alkali (Na_2_Oeq)
CP V ARI ULTRA	Votorantim	L07133708	0.749%
CP V ULTRA RÁPIDO	Lafarge Holcim	F10/09/19-RBOO E3PZ258	0.61%

**Table 4:** List of samples and mixtures produced for expansion test in concrete prisms.

Test	Sample	Mixture (kg)
Long-term method	STL1	1:1.56:2.56:0.45
	STL2	1:1.51:2.61:0.45
	STL3	1:1.41:2.62:0.45
	ALP2	1:1.62:2.39:0.45
	ALP3	1:1.69:2.40:0.45
	DIA	1:1.85:2.76: 0.45
Accelerated method	STL0 St Standard	1:1.41:2.62: 0.45
	STL0 100%	

**Table 5:** Summary of petrographic analysis results.

Samples	Mineralogical Composition (%)					
	Quartz	Muscovite	Opaque	Rutile	Tourmaline	Zircon
STL1	94.0	5.7	0.3	-	Trace	
STL2	95.0	4.6	0.4	Trace		
STL3	94.5	5.0	0.5	Trace		
ALP1	94.5	5.2	0.3	Trace	Trace	
ALP2	94.5	5.0	0.5	-		
ALP3	94.5	5.2	0.3	-		

**Table 6:** Summary of expansion results and SEM analysis.

Samples	Mortar Bars Accelerated Method	Concrete Prisms Long-Term Method	Concrete Prisms Accelerated Method	SEM
STL0	Potentially Reactive	-	Potentially Reactive	Reactive
STL1	Potentially Innocuous	Potentially Innocuous	-	-
STL2	Potentially Innocuous	Potentially Reactive	-	-
STL3	Potentially Innocuous	Potentially Reactive	-	-
ALP1	Potentially Innocuous	-	-	-
ALP2	Potentially Reactive	Potentially Reactive	-	Reactive
ALP3	Potentially Innocuous	Potentially Reactive	-	-
DIA	Potentially Reactive	Potentially Innocuous	-	Reactive

## Data Availability

Not applicable.
